# Critical care nurses’ experiences of ethical challenges in end-of-life care

**DOI:** 10.1177/09697330241252975

**Published:** 2024-05-22

**Authors:** Lena Palmryd, Åsa Rejnö, Anette Alvariza, Tove Godskesen

**Affiliations:** 7643 Marie Cederschiöld University; Karolinska University Hospital; University West; Skaraborg Hospital Skövde; Skaraborg institute for Research and Development; 7643 Marie Cederschiöld University; Stockholms Sjukhem; 1786 Nord University; 8097 Uppsala University; 7643 Marie Cederschiöld University

**Keywords:** End-of-life care, ethical challenges, intensive care, interpretive description, nursing care, qualitative research

## Abstract

**Background:**

In Swedish intensive care units, nine percent of patients do not survive despite receiving advanced life-sustaining treatments. As these patients transition to end-of-life care, ethical considerations may become paramount.

**Aim:**

To explore the ethical challenges that critical care nurses encounter when caring for patients at the end of life in an intensive care context.

**Research design:**

The study used a qualitative approach with an interpretive descriptive design.

**Research context and participants:**

Twenty critical care nurses from eight intensive care units in an urban region in Sweden were interviewed, predominately women with a median age of fifty-one years.

**Ethical considerations:**

This study was approved by The Swedish Ethics Review Authority.

**Findings:**

Critical care nurses described encountering ethical challenges when life-sustaining treatments persisted to patients with minimal survival prospects and when administering pain-relieving medications that could inadvertently hasten patients’ deaths. Challenges also arose when patients expressed a desire to withdraw life-sustaining treatments despite the possibility of recovery, or when family members wanted to shield patients from information about a poor prognosis; these wishes occasionally conflicted with healthcare guidelines. The critical care nurses also encountered ethical challenges when caring for potential organ donors, highlighting the balance between organ preservation and maintaining patient dignity.

**Conclusion:**

Critical care nurses encountered ethical challenges when caring for patients at the end of life. They described issues ranging from life-sustaining treatments and administration of pain-relief, to patient preferences and organ donation considerations. Addressing these ethical challenges is essential for delivering compassionate person-centered care, and supporting family members during end-of-life care in an intensive care context.

## Introduction

Intensive care comprises highly specialised nursing and medical competence, as well as advanced technology, to meet the complex needs of seriously ill patients with critical conditions necessitating advanced life-sustaining treatments.^1^ For patients where there is no possibility of survival, the care provided transitions from having curative intentions to instead focussing on relieving symptoms and promoting well-being at the end-of-life. During end-of-life care, ethical challenges may arise for critical care nurses (hereafter referred to as CCNs), for example, when patients’ preferences regarding continued care cannot be identified,^2^ or when there are disagreements in decisions about patients’ care.^3^

## Background

Medical and technological innovations in intensive care have led to a dramatic improvement in patients’ survival over the past decades.^4^ Nevertheless, in 2022, about nine percent of patients in Swedish intensive care units (ICUs) died, often while receiving a high level of treatment.^5^ For patients where further aggressive treatment is unlikely to provide meaningful benefits, the focus shifts to providing comfort care. End-of-life conversations with patients and their family members should then be initiated concerning their wishes regarding continued care, taking into account the patient’s condition and needs.^6^ These end-of-life conversations might include decisions about withholding or ending life-sustaining treatments and interventions.^2,7^

End-of-life care should be guided by a palliative care approach, which aims to relieve symptoms, improve the quality of life of patients with life-threatening illnesses, as well as offer support to family members.^8^ In intensive care, CCNs bear the primary responsibility of being present around the clock. CCNs are professionals who are expected to provide continuous monitoring, perform treatments, administer medication, collaborate with medical teams, offer emotional support, and respond to emergencies.^9^ CCNs’ specific competence is a prerequisite for making adequate observations, assessments, planning and measures in patient care.^10^ CCNs closely monitor the patient’s vital signs and with their constant presence they should be able to interpret any signs of deterioration or changes in the patient’s condition, for example, pain.^11^ In the changes that CCNs perceive, they may also acknowledge and interpret facial expressions and other signs that lead them to understand that a patient is approaching death.^10^

There is a risk that nurses experience ethical challenges in care, which mainly arise when they fail to properly respect the patient’s privacy when performing nursing actions. Ethical challenges can also arise when they need to perform care interventions that has been decided by others, foremost physicians, and the decision is in conflict with the nurses’ own values and norms.^12^ An important part of CCN’s work in end-of-life care is to create an environment that is peaceful and dignified for patients nearing end of life, by reducing the technical life support devices.^13^ By creating an environment where the patients are comfortable and free from pain and anxiety, the CCNs strives to contribute to an increased quality of life.^14^ Supporting family members is also of importance, especially in situations when receiving information about a patient’s life-limiting diagnosis, which may lead to shock and emotional distress.^15,16^ Family members then need to adapt to, and understand, the critical situation and deal with the threat of death and loss.^16^ The involvement of family members is crucial when patients do not survive and if organ donation after brain death^17^ or circulatory death^18^ becomes relevant. Family members therefore often contribute to ensuring that the wishes of a patient are met, and their values and preferences protected.^19^

Facing ethical aspects of care can be related to nurses international code of ethics, ICN,^20^ which outlines that nurses are responsible for creating an environment that respects the human rights, values, customs, religious and spiritual beliefs of individuals and families. In their daily roles in ICUs, CCNs act as advocates for the patients. They are responsible for upholding a culture that not only promotes ethical conduct, but also encourages open dialogue.^20^ In the ICU setting, CCNs responsibilities are closely linked to the ICNs ethical code, which emphasizes the importance of personal reflection in the delivery of care. The practice of nursing is deeply personal, and good nursing is ‘more than competent performance of a number of caring activities’.^21^ The nurse’s character also significantly influences their care.^22^ To navigate the ethical challenges in the ICUs effectively, nurses can draw upon the perspectives offered by Gastmans^23^ which advocate for a fundamental ethical approach to nursing. This approach is characterized by a ‘caring behaviour’, where competence, skills, and attitudes converge to define good care. Such behaviours foster a therapeutic relationship between the nurse and the patient, where the nurse’s moral sensitivity and attentiveness play a crucial role in identifying and addressing the vulnerabilities in patients. By embodying this morally sensitive attitude, CCNs not only respond to the immediate needs of care but also engage in a deeper, ethical connection with their patients.

A previous review studying nurses’ ethical reasoning and behaviour in mixed care contexts, which also included ICUs, showed that nurses’ ethical practice is a complex process of reasoning and decision-making, and is influenced by personal and contextual factors.^24^ From previous studies on ethical challenges for CCNs in end-of-life care in ICUs, Jensen et al.^2^ describe that the end-of-life decision-making process can be changed or postponed, caused by a lack of continuity of healthcare staff with different perspectives in patient care. Further, ethical challenges for nurses have been identified by Fridh et al.^14^ when cardiopulmonary resuscitation was performed with family members nearby, or when patients died without close family members present. In a systematic review by Metaxa et al.,^25^ it was found that very few of the studies reported on ethicists consultations concerning care at the end of life for patients in ICU. This highlights a significant gap in the literature, and the authors advocate for further research into ICU-based end-of-life care. In the existing literature, knowledge is limited about the complex ethical challenges faced by CCNs in end-of-life care in ICUs. Since 2009, this need has been noticed, and still exists to a large extent.^24^ There is therefore a need for research, to expand the knowledge of ethical challenges in the ICUs, which underlines the need for this study. Through an exploration of the complexity of care, this study seeks to amplify ethical awareness and to promote patient-centred care within the end-of-life context, with particular emphasis on addressing ethical challenges within the ICU setting.

## Aim

To explore the ethical challenges that CCNs encounter when caring for patients at the end of life in an intensive care context.

## Method

### Research design

The study used a qualitative approach with an interpretive descriptive design.

### Research context and participants

The participants were recruited from eight ICUs that cared for adult patients in an urban region in Sweden. Of the ICUs, five focused on general intensive care for a wide range of medical conditions, and three focussed on highly specialized care in neuro-, trauma- and thoracic-intensive care. All ICUs had six to ten beds, usually in single or two-bedded rooms and sometimes multi-bedded rooms. The ICUs had at least two intensive care physicians in attendance around the clock, one of whom was a senior physician. In Swedish ICUs, each patient is assigned a CCN who is responsible for the nursing care and for care plans being established and updated. The goal is to offer patients highly competent nursing care and provide individual support for family members based on their needs. All ICUs had an unlimited visiting policy which allowed family members unrestricted access to patients. Furthermore, family members were provided with a dedicated common room in the ICUs where they could rest and eat.

The inclusion criteria for participation in the study were being a CCN with a minimum of one year’s professional experience in an ICU. A total of twenty CCNs participated in the study. Of these, eighteen were women and two men, with work experience ranging from one to thirty-five years, median eleven years.

### Procedure and data collection

The administrative head managers at the eight ICUs were contacted regarding approval for the study. Following approval, the head nurses acted as liaisons and assisted in coordinating an information session for the CCNs led by the first author (LP). This was, however, only feasible at two of the participating ICUs with the others citing heavy workloads. All units received written information about the study, including contact details to LP to be given to eligible CCNs. Those interested in participating in the study contacted LP to schedule an interview. An interview guide with open questions was designed for the study based on the literature and the research group’s clinical experience in intensive care and palliative care.^26^ The interview guide focused on ethical challenges for CCNs in their daily work with dying patients and family members (Table 1). Three pilot interviews were conducted to ensure that the interview guide was comprehensive and understandable. Since these resulted in only minor adjustments (two similar questions were merged into one), all three pilot interviews were included in the analysis.

**Table 1.** Interview guide.

Ethical competence	- What is ethics for you in your role as a nurse? - Do you feel confident with your knowledge and skills to deal with challenging situations?
End-of-life care in ICU	- What skills and knowledge do you think nurses need to provide good care to a dying patient? - What do you think is important when caring for patients at the end of life? - Are there situations when it feels ethically problematic? - If you think back, what do you think has been most challenging when providing care to patients at the end of life?
Family members’ participation, support needs and wishes	- Are close family members usually involved in the decisions that are made? - Have you ever felt that the role of family members is ethically problematic? - What do you think family members might need for support?
Support in work	- Do you discuss ethical issues that arise in connection with end-of-life care? - Do you experience any obstacles to discussing ethical issues?

All interviews were conducted by LP from October to December 2021 in secluded rooms near the wards during the CCNs’ working hours. The interviews began by asking the CCNs about their perception of ethics in their professional role as a nurse, followed by questions regarding their experiences of ethical challenges in intensive care. The CCNs themselves defined what ethics means to them, and what would constitute an ethical challenge. Questions were then posed concerning their daily work with dying patients and their family members. The interviewer followed up responses with probing questions, such as: ‘Can you tell more about that?’ When necessary, CCNs were given time to reflect on their thoughts in silence before responding. All interviews were audio-recorded and transcribed verbatim.

### Data analysis

The analysis was inductive, using the specific methodological phases of Interpretive Description (ID) to guide the process.^27,28^ LP started by repeatedly listening to the recorded interviews while reading the transcripts to become familiar with the data, and making memos to document reflections on the underlying meaning of the content. In the second phase, units of text that captured stories closely aligned with the study aim were selected for further analysis. This selection was carried out through collaborative dialogue between LP and last author (TG). In the third phase, TG had analysed six interviews with the intention of interpreting and condensing the selected text units working in conjunction with LP. Throughout this process, great emphasis was placed on retaining the essence of the stories and ensuring that the text units were abstracted to a general level. Thereafter, LP proceeded to analyse the remaining selected text units. This phase ended with collaborative reflection involving all researchers, where text units were interpreted and the further coding process was discussed. In the fourth phase, LP and TG began organising the interpreted data into segments with similar content which then formed the codes, for example, ‘Difficult to know the limits when dosing palliative medications’. In the interpretive phase, patterns of similarities and variations in the data were identified. LP then continued organising the remaining data units. At the end of this phase, all authors met and reflected on the codes and engaged in discussions about potential themes to be further processed. Following this, the fifth phase involved the development of themes derived from the synthesised codes, drawing upon the themes that had been collectively discussed by all authors. Throughout the process, variations in the participants’ experiences were sought to gain a broader insight into the findings. The authors discussed the varied experiences of CCNs further and reached consensus on the final analysis.

### Rigour

Rigour was sought by achieving trustworthiness according to Lincoln and Guba’s framework.^29^ Credibility has been strengthened by the fact that all participants were specialist nurses in intensive care with varying experience of working in different ICUs. Credibility was further enhanced by the composition of the research group, which includes four authors, all RNs, with diverse experience from different contexts. Dependability is increased by the researchers having previous experience of research in intensive care, specialised palliative care, acute stroke care and oncology care. LP, who also works as a CCN, has been attentive to the need to maintain a neutral approach during the interviews. The research group held frequent meetings, ensuring an open dialogue and reflected on the analysis process. Since attention to rigour is crucial in ID, the researchers have jointly reflected on alternative choices in order to reach confirmability.^30^ In their reflections, the research team strove to avoid interpretations not supported by the data.^27^ The thorough description of the analysis with detailed verbatim quotations aims to give the reader the possibility of assessing the trustworthiness. The findings concerning the ethical challenges in end-of-life care should be transferable to different care contexts, both to similar intensive care contexts but also to other care settings where palliative care may be relevant.^29^

### Ethical considerations

The Swedish Ethical Review Authority approved the study, Dnr 2021-03364. The study was conducted in agreement with the Swedish Ethical Review of Research Involving Humans Act^31^ and the General Data Protection Regulation.^32^ None of the authors in the research group have held formal positions of superiority to the participants. The participants received written information about the study before the interviews. They were also informed that participating in the study was voluntary and that they could withdraw their participation at any time without explanation. The informants were guaranteed that confidentiality would be maintained and individuals could not be identified since the data would be coded and reported at group level. They were also informed that only LP had access to the codelist, which would be stored securely and separated from the data.

## Findings

The findings are described through three themes: *Focusing on relieving suffering; Differing or unknown preferences, norms and values;* and *Considerations of organ donation.*

### Focusing on relieving suffering

A recurring challenge described by the CCNs was the dilemma of providing care for dying patients whose life-sustaining treatments persisted at the physician’s initiative, despite the patients not showing signs of improvement and having a low probability of survival. The CCNs described the ethical challenge as two-fold. First, the CCNs worried that patients could experience prolonged suffering while being deprived of the right to die. Second, they were also concerned that when curative treatments persisted, family members were being given false hope that the patients would survive. As a result, CCNs experienced that family members were unable to support the patients towards the end of life. Without this false hope they might have gained an earlier awareness of the patient’s approaching death. When family members realised late in the illness trajectory that the patient was about to die, their hope turned to despair. This was often considered complex and challenging for the CCNs. As a consequence, death could be experienced as sudden even though the dying process had been ongoing for some time.

*…now we‘ve done too much and it doesn’t feel right. Partly because it gives family members a kind of hope that things will go well, partly because people are subjected to painful examinations and treatments that might not make them better anyway. (CCN 9)*

The CCNs described that an integral part of end-of-life care involved creating a comfortable environment where patients experienced relief from pain and suffering. A recurring difficulty for the CCNs was often balancing the dosage of pain relief, which was based on the patient’s individual pain level. The ethical challenge in this situation was the potential harm associated with administrating too high doses, which in a worst scenario could inadvertently hasten the patient’s death. In situations like these, the CCNs often felt that their professional experience facilitated making assessments. Despite the difficulty of balancing the dosage of pain-relieving medications, the CCNs strived to ensure optimal comfort for the patients at the end of life.

*I’ve found it difficult, finding the balance between providing good pain relief and anxiety relief versus not tipping things over the edge. I’ve found this balance difficult.* (CCN 19)

Another challenge regarding patients’ comfort could arise when there were disparities in the perception of patients’ pain between the CCNs and family members. The ethical challenge for CCNs occurred in situations where family members perceived patients as suffering and in need of pain-relieving medication, while CCNs evaluated the patients’ pain relief to be adequate and consequently hesitated to administer additional medication. A CCN clarified with the comment *‘Often, family members can say things like ‘Can’t you give a bit more? And can’t you give some more?’* (CCN 19). There were also concerns among CCNs that if they accommodated family members requests for more pain-relieving medications, they would risk giving medication that shortened the patients’ lives.

### Differing or unknown preferences, norms and values

The CCNs often experienced challenges when patients’ individual preferences were unknown, leading to uncertainty about how the care should proceed. The ethical challenge increased when patients were unconscious due to illness and sedation, rendering direct communication impossible. CCNs felt less challenged when family members were present as they could often act as proxies and convey what they believed to be the patient’s wishes.

Most patients were unconscious during their last moments of life. However, some patients were conscious and could communicate their preferences right to the end. The CCNs reported that ethical challenges could arise when conscious patients communicated preferences that conflicted with the CCNs’ values. This conflict might arise in instances where patients expressed a desire to discontinue life-sustaining treatment, such as breathing support with a ventilator, while from their professional experience, the CCNs believed that proper care could potentially extend the patient’s lifespan significantly. Despite having differing perceptions, the CCNs strove for patients’ preferences to be prioritised and for patients to feel empowered to make decisions about their care.

*You will need a ventilator for the rest of your life/…/ [patient replies] ‘No, I don´t want that. I want you to remove the ventilator, because I don’t want it. I don’t want to be dependent on a ventilator for the rest of my life, then I’d rather die.’ Then you end up in an ethical dilemma, I think.* (CCN 3)

Based on the CCNs experiences of end-of-life care, it was customary in certain families to shield dying patients from the knowledge of their impending death. This custom often posed a challenge for the CCNs, since Swedish healthcare regulations uphold the patient’s entitlement to receive information about their diagnosis and prognosis. When patients right to information was challenged through family members wanting to withhold information about, for example, a poor prognosis, the CCNs experienced an ethical challenge. This was because the patient’s autonomy was violated since they were being deprived of the opportunity to receive information about their condition and be involved in decisions about their care.

*... then dad, or whoever it is, shouldn’t be allowed to find out how things really are because he shouldn’t have to worry about that.* (CCN 9)

Another challenge for CCNs occurred when family members expressed wishes to transport patients to their country of birth in order to die surrounded by their entire family. This presented an ethical challenge for the CCNs because, on the one hand, if the patients travelled to their country of birth, this might risk them dying during the journey, which would deprive them of the opportunity to share their last moments with the entire family present. On the other hand, if the patients choose not to travel, they miss out on the opportunity to die surrounded by their family members; travelling might have allowed them to achieve their wishes.

*...and he wants his mother to go back to their country of birth so they can meet. It can be problematic trying to make him understand that transporting a dying woman to another country is an impossibility.* (CCN 5)

Providing end-of-life care based on the patients’ individual preferences, whether known or unknown, was often described as a major challenge, as was family members questioning the healthcare regulations.

### Considerations of organ donation

Caring for a deceased person’s body as a potential organ donor was often described by the CCNs as challenging. The ethical challenge was based on their responsibility for the care of a dead person’s body with organ-preserving treatment. A central concern for CCNs was that if the potential organ donor suffered a cardiac arrest, they were unsure if they should initiate cardiopulmonary resuscitation. The CCNs questioned whether this would be in the best interest of the donor or the potential organ recipients.

*Donation has already been decided and the patient has a cardiac arrest/…/what should we do, and for whose sake, for the sake of the patient, the organs, those who are waiting to receive the organs?* (CCN 7)

The CCNs noted that some patients had planned and documented their preferences regarding organ donation in advance. As these patients neared the end of life and donation became relevant, their family members were informed of the patients’ expressed wishes. However, despite the patients’ explicitly stated preferences, there were family members who neither embraced nor endorsed these wishes. These situations represented an ethical challenge for the CCNs as they observed the patient’s autonomy being violated when the patient’s preferences were not paramount in the decision-making process concerning organ donation.

*...perhaps there’s some doubt, it can be about everything, eh, that the patient has said ‘yes’ to donation but the family members say ‘no’.* (CCN 14)

After organ donations were completed, follow-up conversations were often held with the family members, giving them the opportunity for their remaining questions about the donation process to be answered by CCNs and physicians. Within these conversations, the CCNs perceived that some family members could be unsure about when the patient had actually died. For the CCNs, an ethical challenge could arise if they discovered that the family members were resistant to the information given due to the crisis they were going through.

*...that the patient has suffered a total cerebral infarction, they seem to have missed that. They maybe thought that, well, death occurred during [donation] surgery. (CCN 8)*

Caring for a potential organ donor, while being attentive to and supporting the needs of family members, was often considered by the CCNs to be one of the most complex parts of care.

## Discussion

This study explored the ethical challenges that CCNs encounter when caring for patients at the end of life in an ICU context. The findings are described through three themes: Focusing on relieving suffering; differing or unknown preferences, norms and values; and Considerations of organ donation. Major ethical challenges included the withdrawal of life-sustaining treatments for patients with a limited chance of survival, as well as managing the risk of pain medications inadvertently accelerating death. These challenges involved situations where patients wished to discontinue treatment despite the prospect of a longer life, family members’ protective instincts regarding withholding crucial health information, and the complexities of caring for a deceased individual for potential organ donation.

The CCNs encountered ethical challenges when life-sustaining treatments persisted despite patients having limited chances of survival. This can lead to CCNs experiencing significant moral distress, especially when life-saving measures are initiated in patients with a low probability of survival, or when no physician makes the decision to withdraw respiratory support.^33^ While CCNs perceive these situations as ethically challenging, they acknowledge that physicians may not always share their perspectives and might view CCNs as pessimistic about patients’ chances of recovery. Additionally, this study highlights instances where CCNs observed family members being given false hope of the patient’s survival, which can prevent them from adequately preparing for the impending death. However, it is also worth noting that CCNs may perceive the persisted treatments to be beneficial to family members, since it can provide them with time to say a final farewell.^33^

Administering pain-relieving medications that may inadvertently hasten the death of patients was another common ethical challenge for CCNs. Patients in various care settings, are at risk of experiencing pain with symptoms of anxiety, confusion and nausea at the end of life.^34^ In ICUs, CCNs have perceived that patients may also experience symptoms such as physical pain, difficulty breathing, and swelling.^35^ Registered nurses (RNs) have reported that they may withhold symptom relief from pain-affected patients due to the fear of unintentionally causing death and facing legal repercussions, job loss or revocation of registration.^36^ However, with family members present, RNs feel they can administer more generous amounts of pain medication to patients nearing the end of lives.^37^

The CCNs faced ethical challenges when patients expressed wishes to discontinue life-sustaining treatments, even though their life could be prolonged with proper care. Decision-making concerning the initiation, continuation or withdrawal of these treatments extends beyond medical decisions; patients’ preferences, incorporating their values, beliefs, and wishes, play a pivotal role.^38^ For the CCNs, these ethical challenges often centre on empowering patients to make decisions that reflect their values, based on psychological, social, environmental and spiritual factors.^39^ One can argue that the CCNs were guided by a moral sensitivity as outlined by Gastmans,^23^ and that they were deeply touched by the suffering of others. By doing so, they can have an ability to see beyond the clinical parameters and understand the complex context of patients’ situations. When faced with ethical challenges, CCNs with high moral sensitivity can perceive the nuances of the situation, understand patients’ values, and make decisions that align with ethical norms. In this way, they can alleviate suffering and offer compassionate care in line with patients’ preferences and wishes.

When family members’ wishes conflicted with healthcare guidelines, CCNs could encounter ethical challenges. This could occur when family members tried to protect a patient by withholding information about a poor prognosis. Previous research^40^ has shown that respecting a patient’s autonomy, while simultaneously acknowledging the family members’ concerns, is a complex task. When requests to withhold information are expressed by family members, healthcare professionals may experience an ethical challenge that involves a delicate balance between family members’ wishes and the patient’s right to information, independence and confidentiality. Providing information to patients about their condition and prognosis is often an important part of end-of-life care, as long as the patient is able to assimilate the information.

Caring for a deceased individual’s body prior to possible organ donation presented ethical challenges due to the CCNs’ duty to preserve the organs. Within this specialised area of care, the CCNs strive to treat the deceased with dignity and respect, which often demands a deeper emotional investment than caring for living patients.^41^ Ethical challenges can arise when family members have not yet been asked about organ donation despite the ICU team being aware of the possibility, or when physicians use medical terms when communicating with family members that the CCNs later need to explain, despite them not having participated in the conversation.^42^ CCNs may also encounter challenges if they are unfamiliar with the process and if medical interventions are perceived as complicated.^43^

### Strengths and limitations

A strength of this study is that participants were recruited from eight ICUs and had extensive work experience from ICU care. The interviews were conducted in a secluded yet familiar setting, promoting a relaxed atmosphere; this likely enriched the depth and range of the ethical challenges shared by the participants. Methodological trustworthiness was ensured by providing clear descriptions of the analysis phases, and by substantiating the analysis with direct quotations as suggested in the methodological framework.^27^ Nevertheless, a possible limitation is that the interviewers background as an experienced CCN fostered trust and rapport, enhancing the credibility of the interviews, it also posed a potential limitation and participants may have assumed certain shared knowledge and not elaborated on certain points, which could affect the breadth of the data collected. Another limitation may be that the gender distribution of the participants is uneven, which is not unlike the actual distribution. With only two men participating in the interviews, there may be a lack of a male perspective in the results.

### Clinical implications and recommendations for future research

This study illuminates ethical challenges that CCNs might encounter in end-of-life care, and how challenges can be understood and responded to. These insights can serve as a foundation for discussions about ethical challenges in clinical practice and guide informed decision-making. Attention given to these findings can enhance patient care and support CCNs in navigating complex ethical situations. Based on these findings, future research may include a more innovative approach, such as identifying nurses’ educational needs and co-designing or implementing educational interventions to support CCNs with ethically sound practice. To support and improve end-of-life care, according to Metaxa et al.,^25^ future research should give priority to multifaceted educational initiatives and the integration of specialized palliative care teams in ethical consultations.

### Conclusion

In summary, CCNs encountered significant ethical challenges when caring for patients at the end of life. What our study found is that these challenges encompass decisions about withdrawing life-sustaining treatments for patients with limited survival prospects and balancing symptom relief management without hastening death. The challenges extended to situations where patients desired treatment cessation despite the chance of a longer life, family members striving with safeguarding sensitive health information, and the process of posthumous care in organ donation. Our findings imply that addressing these ethical challenges is essential for providing compassionate, person-centred care, and supporting family members during end-of-life care in an intensive care setting.

## Appendix

### Abbreviations

CCN: Critical Care Nurse
ICU: Intensive Care Unit
ID: Interpretive Description
RN: Registered Nurse
